# Novel Personalized Cancer Vaccine Using Tumor Extracellular Vesicles with Attenuated Tumorigenicity and Enhanced Immunogenicity

**DOI:** 10.1002/advs.202308662

**Published:** 2024-04-26

**Authors:** Jihoon Han, Seohyun Kim, Yeong Ha Hwang, Seong A Kim, Yeji Lee, Jihong Kim, Seongeon Cho, Jiwan Woo, Cherlhyun Jeong, Minsu Kwon, Gi‐Hoon Nam, In‐San Kim

**Affiliations:** ^1^ KU‐KIST Graduate School of Converging Science and Technology Korea University Seoul 02841 Republic of Korea; ^2^ Chemical and Biomedical Integrative Research Center Korea Institute of Science and Technology (KIST) Seoul 02792 Republic of Korea; ^3^ Department of Research and Development ShiftBio Seoul 02751 Republic of Korea; ^4^ Research Animal Resource Center Korea Institute of Science and Technology (KIST) Seoul 02792 Republic of Korea; ^5^ KHU‐KIST Department of Converging Science and Technology Kyung Hee University Seoul 02447 Republic of Korea; ^6^ Department of Otolaryngology Asan Medical Center University of Ulsan College of Medicine Seoul 05505 Republic of Korea; ^7^ Department of Biochemistry and Molecular Biology Korea University College of Medicine Seoul 02841 Republic of Korea

**Keywords:** cancer vaccine, immunotherapy, tumor extracellular vesicles, verteporfin

## Abstract

Cancer vaccines offer a promising avenue in cancer immunotherapy by inducing systemic, tumor‐specific immune responses. Tumor extracellular vesicles (TEVs) are nanoparticles naturally laden with tumor antigens, making them appealing for vaccine development. However, their inherent malignant properties from the original tumor cells limit their direct therapeutic use. This study introduces a novel approach to repurpose TEVs as potent personalized cancer vaccines. The study shows that inhibition of both YAP and autophagy not only diminishes the malignancy‐associated traits of TEVs but also enhances their immunogenic attributes by enriching their load of tumor antigens and adjuvants. These revamped TEVs, termed attenuated yet immunogenically potentiated TEVs (AI‐TEVs), showcase potential in inhibiting tumor growth, both as a preventive measure and a possible treatment for recurrent cancers. They prompt a tumor‐specific and enduring immune memory. In addition, by showing that AI‐TEVs can counteract cancer growth in a personalized vaccine approach, a potential strategy is presented for developing postoperative cancer immunotherapy that's enduring and tailored to individual patients.

## Graphical abstract

Simultaneously inhibiting the YAP protein and autophagy while inducing ICD on tumor cells leads to the production of attenuated yet immunogenically potentiated TEVs (AI‐TEVs). AI‐TEVs effectively activate tumor‐specific immunity and can be used as a prophylactic and post‐operative therapy to prevent cancer recurrence. They can also serve as the basis for personalized cancer vaccines by utilizing autologous tumor cells.

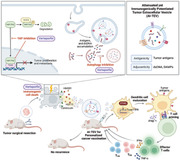

## 1 Introduction

Despite recent advances in surgical techniques and novel cancer therapeutic strategies, the risk of cancer recurrence remains high, posing a threat to countless patients. Therefore, it is necessary to develop a fundamental approach to eliminate residual tumors and prevent cancer recurrence by inducing systemic antitumor responses and retaining long‐term memory. Personalized cancer vaccination is one of the most promising strategies for eliciting and amplifying tumor‐specific immune responses designed individually, using a variety of antigens prepared directly from the patient's tumor tissues.^[^ ^1^ ^]^ However, to develop a successful cancer vaccine, several requirements need to be met, such as an optimal source of tumor antigens and their combination with immune adjuvants, all of which must be formulated in an appropriate delivery platform.^[^ ^2^ ^]^ While there have been numerous efforts to develop personalized cancer vaccines over several decades, most have shown unsatisfactory clinical outcomes owing to the failure to meet these factors.^[^ ^3^ ^]^

Tumor‐derived extracellular vesicles (TEVs) are lipid bilayer particles released from tumor cells into the extracellular environment; they consist of various vesicles, most notably exosomes, apoptotic bodies, and microvesicles.^[^ ^4^ ^]^ They are known to have a unique cargo profile that reflects the characteristics of the original tumor cells, such as proteins, lipids, and nucleic acids, and as a result can influence the functions or phenotypes of recipient cells.^[^ ^5^ ^]^ Numerous studies have shown that TEVs carry tumor antigens, including tumor‐associated antigens and tumor‐specific antigens, which are considered the most important components of an antitumor vaccine.^[^ ^6^, ^7^ ^]^ Moreover, TEVs are natural delivery platforms, indicating their ability to successfully deliver antigenic materials.^[^ ^8^, ^9^ ^]^ Considering these advantages, TEVs have gained increasing attention owing to their tremendous potential to serve as tumor antigens and evoke tumor‐specific immune responses. However, TEVs are known to be involved in immune‐suppressing and tumor‐promoting pathways, making them unsuitable for use in anticancer therapies.^[^ ^10^, ^11^ ^]^ Therefore, to utilize TEVs as an effective cancer vaccine platform, they need to be combined with adjuvants to induce sufficient immune responses as well as a method to silence the tumor‐promoting characteristics of TEVs.

**Figure 1:**
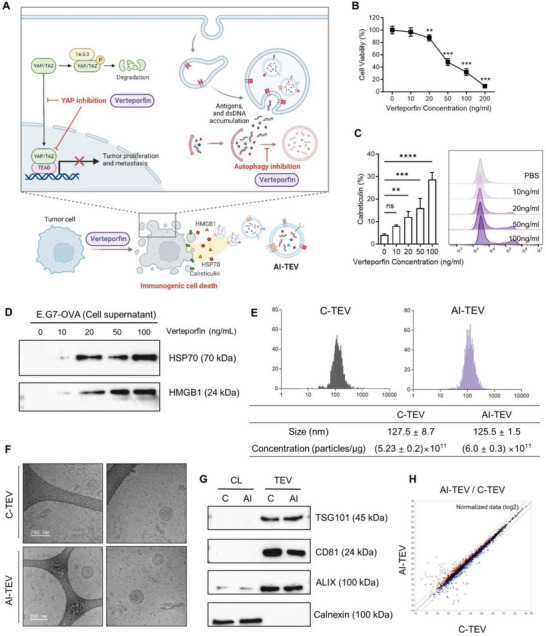
Verteporfin induces ICD in E.G7‐OVA cells, generating AI‐TEVs that are proteomically distinct from C‐TEVs. A) Schematic illustration for generation of AI‐TEVs: Verteporfin treatment attenuates pro‐tumorigenic properties of tumor cells while simultaneously increasing immunogenicity, reflecting these properties on secreted TEVs. B) E.G7‐OVA cells were cultured in with different concentrations of verteporfin for 24 h. Total viable cells were measured with a CCK‐8 assay. (*n* = 8 per group) C) E.G7‐OVA cells were cultured with verteporfin for 24 h, then analyzed via flow cytometry for calreticulin expression on the surface of cell membrane‐gated PI^−^ intact cells. (n = 4 per group) D) Western blotting of HSP70 and HMGB1 released in conditioned media of E.G7‐OVA cells 24 h after verteporfin treatment. E) Size distribution and particle number of TEVs, as assessed using nanoparticle tracking analysis. (*n* = 4 per group) F) Cryogenic transmission electron microscopy image of C‐TEVs and AI‐TEVs (scale bar: 200 nm). G) Western blotting of cell lysates and TEVs detecting EV markers TSG101, CD81, and Alix, along with a negative marker, calnexin. H) Scatter plot comparing the proteomic cargo of C‐TEVs and AI‐TEVs; the dotted lines indicate a two‐fold difference between the samples. One‐way ANOVA followed by Tukey's posthoc test was calculated using GraphPad PRISM (^**^ *p* < 0.01, ^***^ *p* < 0.001, ^****^ *p* < 0.0001). Data are presented as the mean±SD.

Verteporfin is an Food and Drug Administration‐approved drug with the trade name Visudyne, most commonly used as a photosensitizer for photodynamic therapy but also known to have other mechanisms in various diseases.^[^ ^12^, ^13^ ^]^ First, it is a potent Yes‐associated protein (YAP) inhibitor, disrupting the interaction between YAP and TEA/TEF‐domain transcription factors (TEAD) and degrading YAP in the cytoplasm.^[^ ^14^, ^15^ ^]^ The YAP–TEAD complex is a master regulator of the Hippo pathway that regulates cell proliferation, survival and migration, making YAP an oncogene in various tumor types.^[^ ^16^, ^17^, ^18^, ^19^, ^20^ ^]^ Several studies have shown that YAP inhibition attenuates the tumorigenic functions of tumor cells.^[^ ^21^ ^]^ This understanding prompted the hypothesis that verteporfin‐mediated YAP inhibition might reduce the tumorigenicity of TEVs. Morever, verteporfin‐mediated YAP‐dependent cell death has been confirmed to be immunogenic, indicating the potential of YAP inhibition to mediate immune responses by exploiting immunogenic molecules through immunogenic cell death (ICD).^[^ ^22^ ^]^ ICD is a promising mode of cancer cell death where dying cells show increased antigenicity and adjuvanticity, activating antigen‐specific immune responses and thereby inducing potent adaptive immune responses.^[^ ^23^, ^24^ ^]^ Therefore, if verteporfin treatment induces ICD in tumor cells, the cells would show heightened antigenicity and adjuvanticity, which would be reflected in their secreted TEVs that would also be immunogenically potentiated. Second, verteporfin is a well‐recognized autophagy inhibitor, primarily by crosslinking p62, a crucial scaffold protein in the autophagy and thereby preventing autophagosome formation.^[^ ^13^ ^]^ Autophagy regulation is not only associated with the cytoplasmic accumulation of damaged dsDNA, followed by their subsequent transport into excreted EVs, but also an increase in the presentation of tumor antigens.^[^ ^25^, ^26^, ^27^ ^]^ The EV‐loaded DNA fragments can activate the cGAS‐STING pathway in receiving dendritic cells (DCs), initiating a strong immune response.^[^ ^26^, ^28^ ^]^ Therefore, we hypothesized that because verteporfin inhibits both autophagy and YAP protein in tumor cells and simultaneously induces ICD, it could cause tumor cells to secrete TEVs containing high amounts of dsDNA and other immunogenic molecules, such as tumor antigens and danger‐associated molecular patterns (DAMPs), while negating their original pro‐tumorigenic attributes (**Figure** **1**A).

This study aimed to develop attenuated yet immunogenically potentiated TEVs (AI‐TEVs) utilizing the mechanism of verteporfin action in murine models. Moreover, we validated the generation of AI‐TEVs and their effectiveness in therapeutic and prophylactic vaccination models, as well as cancer recurrence and personalized vaccination models.

## 2 Results

### 2.1 Generation and Characterization of AI‐TEVs

To determine the appropriate conditions for generating AI‐TEVs, we treated three types of cancer cells with various doses of verteporfin in serum‐free media and assessed the cell viability. E.G7‐OVA cells are derivatives of EL4 mouse lymphoma cells that carry a complete copy of chicken ovalbumin (OVA) mRNA. Murine oral cancer 2 (MOC2) cells represent an aggressive form of mouse oral squamous cell carcinoma, while 4T1 cells are triple‐negative, highly invasive and malignant murine breast cancer cells. The lowest dose of verteporfin that induced notable cell death was 20 ng mL^−1^ for E.G7‐OVA cells and 200 ng mL^−1^ for MOC2 and 4T1 cells, with ≈80% viability for all cell types (Figures 1B; S1A, Supporting Information). The immunogenic nature of these cell deaths was verified based on an increase in DAMPs after verteporfin treatment. The cancer cells showed a dose‐dependent increase in the expression of calreticulin, as well as the release of heat shock protein 70 (HSP70) and high mobility group box 1 (HMGB1) (Figures 1C,D; S1B,C, Supporting Information), which are widely accepted immunogenic markers that define ICD.^[^ ^24^ ^]^ Based on these results, optimum verteporfin dosage to induce adequate ICD without severely affecting cell viability was selected for subsequent experiments (20 ng mL^−1^ for E.G7‐OVA and 200 ng mL^−1^ for MOC2 and 4T1 cells). Tumor cells were incubated in serum‐free media for 24 h with or without verteporfin to generate AI‐TEVs or control TEVs (C‐TEVs), respectively. When TEVs were analyzed via nanoparticle tracking analysis and cryogenic transmission electron microscopy, E.G7‐OVA TEVs were uniformly round shaped with a diameter of approximately 125–127 nm (Figure 1E,F); a similar pattern was observed for MOC2 and 4T1 TEVs (Figure S2A,B, Supporting Information). TEVs were found to express several EV markers, such as tumor susceptibility gene 101 (TSG101), CD81, and Alix, while absent of the negative marker calnexin (Figures 1G; S2C, Supporting Information). As verteporfin inhibits both autophagy and YAP, we anticipated a significant difference in the proteomic cargo between C‐TEVs and AI‐TEVs; this was confirmed through proteomic analysis and is shown in the scatter plot of normalized data (Figure 1H).^[^ ^15^, ^29^ ^]^ In total, 2509 proteins were detected in both samples, with 529 proteins showing a relative increase in AI‐TEVs and 315 proteins showing an increase in C‐TEVs.

### 2.2 Verteporfin Treatment Induces a Difference in Proteomic Cargo of TEVs, Leading to an Attenuation of Pro‐Tumorigenic Properties

**Figure 2:**
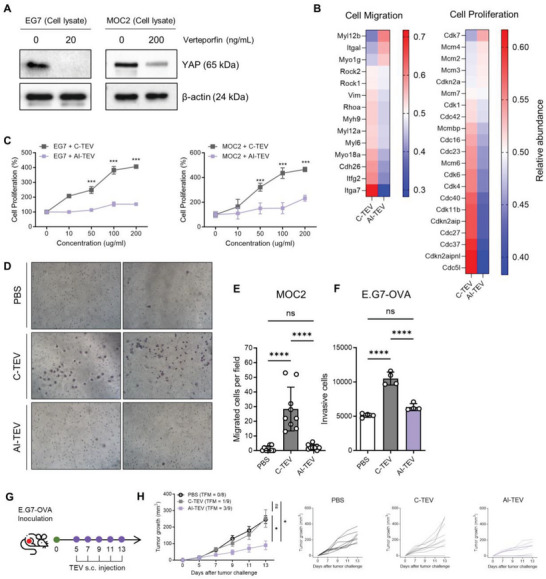
Verteporfin inhibits YAP in cancer cells, leading to the secretion of benign TEVs. A) Western blot analysis of E.G7‐OVA and MOC2 cells treated with verteporfin for 24 h. Cell lysates were subjected to western blot analysis by anti‐YAP and anti‐β‐actin antibodies, demonstrating a decrease of YAP protein in cells. B) Heatmap showing the relative abundance proteins in E.G7‐OVA TEVs, specifically those known to induce cell migration and proliferation, directly downstream of YAP/TAZ Hippo signaling pathway. The color code indicates relative abundance, ranging from blue (low abundance) white to red (high abundance). C) Cell proliferation assay of E.G7‐OVA and MOC2 cells each treated with their respective TEVs in various doses for 24 h. Proliferation of live cells was measured using a CCK‐8 assay, and compared with untreated cells. (*n* = 6–8 per group) D, E) Transwell migration assay of MOC2 cells. Cells were suspended in media containing 1% FBS and placed in the upper chamber of the transwell, while the lower chamber was loaded with media containing 10% FBS. Cells were treated with their respective TEVs in the upper chamber. Migration was observed after 24 h. D) Representative microscopic images of MOC2 cells that have migrated through the transwell (Crystal violet staining. Magnification, X10). E) Number of migrated MOC2 cells were counted using ImageJ software. F) Number of invasive E.G7‐OVA cells in a transwell assay. Since E.G7‐OVA are suspension cells, the number of cells that have invaded to the lower chamber were counted using flow cytometry. G) Therapeutic vaccination schedule of TEVs. H) Average tumor growth curves (*n* = 8–9 per group) and individual tumor growth curves for each group. One‐way ANOVA followed by Tukey's posthoc test was calculated using GraphPad PRISM ((^*^ *p* < 0.05, ^***^ *p* < 0.001, ^****^ *p* < 0.0001). Data are presented as the mean±SD. TFM, Tumor free mice.

As verteporfin is known to disrupt YAP–TEAD interaction and promote YAP degradation in the cytosol, we confirmed that verteporfin treatment reduced YAP protein content in E.G7‐OVA and MOC2 cells (**Figure** **2**A).^[^ ^15^ ^]^ Subsequently, among the total of 2507 proteins revealed in the proteomic analysis of E.G7‐OVA TEVs, careful examination of specific proteins that were directly downstream of YAP‐TEAD signaling was conducted, considering that YAP is a well‐known oncogene that promotes cancer cell metastasis and progression.^[^ ^20^ ^]^ This revealed that AI‐TEVs had a significant decrease in most YAP‐dependent proteins, especially those related to cell migration and proliferation (Figure 2B). This demonstrated that AI‐TEVs carried far less pro‐tumorigenic cargo than C‐TEVs owing to the suppression of YAP. To further demonstrate the attenuated tumor‐promoting properties of AI‐TEVs, we incubated cancer cells with their respective TEVs for 24 h and observed that while C‐TEVs induced a significant increase in cancer cell proliferation, AI‐TEVs did not (Figure 2C). Additionally, the migratory ability of tumor cells stimulated by TEVs was assessed using a transwell migration assay. Microscopic examination revealed that MOC2 cells incubated with C‐TEVs showed significant migration, while cells incubated with AI‐TEVs showed much less migration, with no significant difference between cells treated with phosphate‐buffered saline (PBS) and those treated with AI‐TEVs. E.G7‐OVA cells were also assessed using the same protocol, but because of their non‐adhesive nature, invasive cells were counted in the lower well of the transwell chamber rather than on the membrane (Figure 2D–F). Similar results were observed in 4T1 cells, which also showed verteporfin‐mediated YAP inhibition and consequent differences in proteomic cargo as well as proliferation of TEV‐treated tumor cells (Figure S3A–C, Supporting Information). Owing to the aggressive and metastatic nature of 4T1 cells, a coating of Matrigel was added to the transwell membrane for the invasion assay. Microscopic analysis of 4T1 cells treated with TEVs demonstrated a significant difference in invasion between cells treated with C‐TEVs and AI‐TEVs (Figure S3D,E, Supporting Information). Finally, to verify the attenuation of tumor‐promoting characteristics in AI‐TEVs in vivo, we evaluated their effects as a therapeutic vaccine in murine models. Therapeutic vaccines are another well‐studied form of tumor vaccine designed to inhibit tumor growth and induce long‐term antitumor memory.^[^ ^30^ ^]^ Among E.G7‐OVA tumor‐bearing mice injected with TEVs or PBS (Figure 2G), those injected with AI‐TEVs showed a higher ability to delay tumor growth compared to the other groups, with three out of nine mice being tumor‐free (Figure 2H). While one out of nine mice in the C‐TEV group was also tumor‐free, the remaining eight mice showed tumor growth that was just as fast and aggressive as that in the PBS group, with no significant differences between the two groups. These results verify our hypothesis that, while C‐TEVs cannot hinder tumor growth, AI‐TEVs are attenuated of these properties, thus demonstrating their potential as vaccines that selectively carry the desired qualities of TEVs without their tumor‐promoting effects.

### 2.3 Highly Immunogenic AI‐TEVs Activate the cGAS‐STING Pathway and Promote Maturation in DCs

For TEVs to be effective cancer vaccines, not only do they need to be free from their tumor‐promoting aspects, but they also need adjuvants that can sufficiently stimulate the innate immune system.^[^ ^31^ ^]^ We demonstrated that AI‐TEVs can act as both a vaccine and an adjuvant, triggering a strong immune response. DCs are antigen‐presenting cells that act as mediators between innate and adaptive immunity, and are involved in the direct activation of CD8^+^ cytotoxic T cells. Therefore, to assess the potential of AI‐TEVs, it is critical to evaluate their ability to activate DCs. As verteporfin is widely used as an autophagy inhibitor, we hypothesized that verteporfin leads to the accumulation of tumor antigens and various adjuvant factors in the cytoplasm, especially DAMPs and nucleic acids, and a subsequent increase in their release through TEVs.

**Figure 3:**
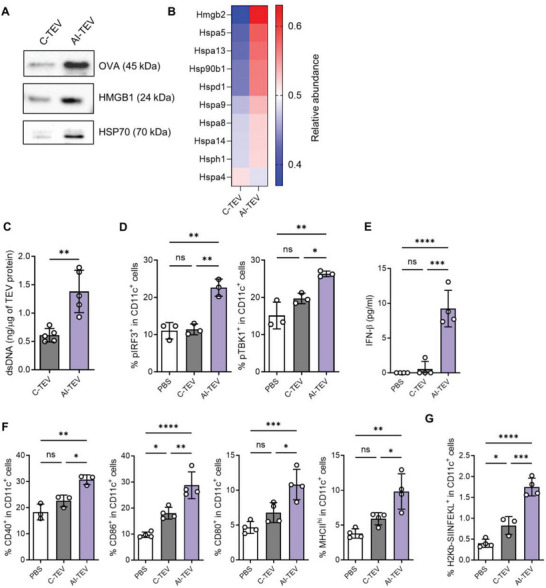
AI‐TEVs are rich in antigenicity and adjuvanticity, therefore have heightened ability to prime dendritic cells. A) Western blot analysis of TEVs detecting ovalbumin (OVA) to represent tumor antigen of E.G7‐OVA cells, and DAMPs (HMGB1, HSP70). AI‐TEVs showed a significant increase in both tumor antigen and DAMPs compared to C‐TEVs. B) Heatmap showing the relative abundance of other various DAMPs in E.G7‐OVA TEVs. Most DAMPs showed a significant increase in AI‐TEVs. C) Quantification of dsDNA in E.G7‐OVA TEVs using QuantiFluor dsDNA system. D) Flow cytometric analysis of pIRF3 and pTBK1 expression in CD11c^+^ BMDCs treated with E.G7‐OVA TEVs for 48 h. E) IFN‐β protein levels in E.G7‐OVA TEV‐treated BMDC supernatants were quantified by ELISA. F) Flow cytometric analysis of DC maturation markers (CD40, CD86, CD80, MHC‐II) in CD11c^+^ BMDCs treated with E.G7‐OVA TEVs for 48 h. G) Flow cytometric analysis of H‐2Kb‐SIINFEKL on surface of BMDCs treated with E.G7‐OVA TEVs for 48 h. One‐way ANOVA followed by Tukey's posthoc test was calculated using GraphPad PRISM (^*^ *p* < 0.05, ^**^ *p* < 0.01, ^***^ *p* < 0.001, ^****^ *p* < 0.0001). Data are presented as the mean±SD.

As expected, immunoblotting of E.G7‐OVA‐derived TEVs revealed a significant increase in ovalbumin (OVA) and DAMPs (HSP70, and HMGB1) in AI‐TEVs (**Figure** **3**A). Moreover, proteomic analysis of TEV cargo showed an increase in most DAMPs in AI‐TEVs compared to that in C‐TEVs (Figure 3B). Previous studies have described that TEVs carrying dsDNA originating from cancer cells activate the cGAS‐STING pathway in DCs.^[^ ^28^ ^]^ A recent study demonstrated that autophagy inhibition in leukemia cells increased cytoplasmic dsDNA accumulation that was subsequently transferred to their EVs, leading to the activation of the cGAS‐STING pathway in bone marrow cells.^[^ ^26^ ^]^ Therefore, we hypothesized that verteporfin‐mediated autophagy inhibition would increase the quantity of dsDNA in the cytoplasm of tumor cells and AI‐TEVs, consequently leading to improved activation of the cGAS‐STING pathway in DCs. Using the QuantiFluor dsDNA System, we demonstrated that AI‐TEVs carried much more dsDNA than C‐TEVs, and E.G7‐OVA‐derived AI‐TEVs showed an almost three‐fold increase in dsDNA compared to C‐TEVs (Figure 3C; Figure S4A, Supporting Information). Thus, we concluded that AI‐TEVs carry high amounts of tumor antigens, DAMPs, and dsDNA.

We then assessed the ability of AI‐TEVs to activate DCs by treating bone marrow‐derived dendritic cells (BMDCs) with TEVs and analyzing the differences via flow cytometry. Notable markers for the cGAS‐STING pathway, phosphorylated interferon regulatory factor 3 (pIRF3), and phosphorylated TANK‐binding kinase 1 (pTBK1), were increased in DCs treated with AI‐TEVs compared to that in other groups (Figure 3D; Figure S4B, Supporting Information). This increase of cGAS‐STING downstream molecules showed a dose dependency to some degree up to 10 µg mL^−1^ of AI‐TEVs (Figure S4C, Supporting Information). A significant increase in IFN‐β release from these DCs was also observed (Figure 3E), further suggesting an effective activation of the cGAS‐STING pathway. The levels of maturation markers for DCs (CD40, CD86, CD80 and MHC‐II) were also elevated (Figure 3F; Figure S4D, Supporting Information), presumably due to the uptake of DAMPs delivered via TEVs and activation of the cGAS‐STING pathway. BMDCs cultured with E.G7‐OVA‐derived TEVs were analyzed for cross‐presentation of OVA (SIINFEKL) peptide and showed a highly significant enhancement of DC cross‐presentation in groups treated with AI‐TEVs (Figure 3G). Overall, these results validate that AI‐TEVs carry sufficient antigens and adjuvants to induce significant activation of DCs, enhancing their cross‐presentation and proving their potential to provoke strong and lasting immune responses.

### 2.4 AI‐TEVs Act as an Effective Prophylactic Vaccine, Prompting Tumor‐Specific Immunity

**Figure 4:**
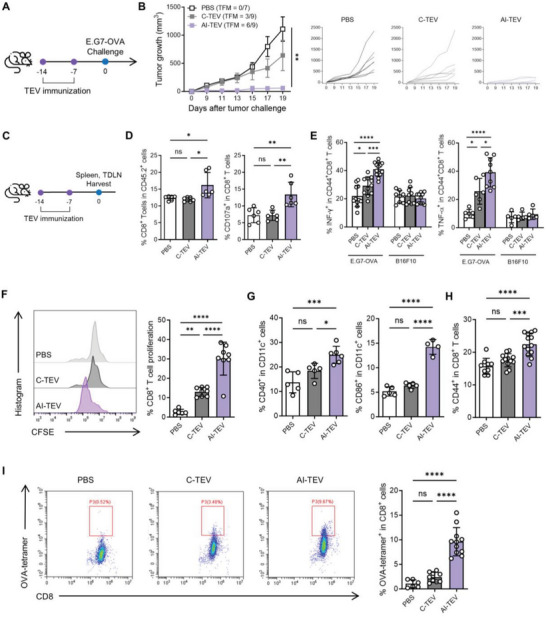
AI‐TEVs act as an effective prophylactic vaccine, inducing tumor‐specific immunity. A) Prophylactic vaccination schedule of E.G7‐OVA‐derived TEVs. B) Average tumor growth curves (*n* = 7‐9 per group) and individual tumor growth curves for each group. C) Scheme of vaccination experiment design for analysis of E.G7‐OVA‐derived TEV‐induced immunity. D) Flow cytometry analyses of the percentage of CD3^+^ CD8^+^ T cells among CD45.2^+^ cells and CD107a^+^ cells among CD8^+^ T cells. E) Splenocytes were stimulated with UV‐irradiated cancer cells for 5 h and analyzed with flow cytometry to identify the percentage of IFN‐γ^+^ and TNF‐α^+^ cells among CD44^+^CD8^+^ cells. F) Splenocytes were stained with CFSE before being stimulated with UV‐irradiated cancer cells for 72 h, then analyzed with flow cytometry for proliferation of CD3^+^CD8^+^ cells. G) Flow cytometry analyses of the percentage of CD40^+^ and CD86^+^ cells among CD11c^+^ cells from TDLNs. H) Flow cytometry analyses of the percentage of CD44^+^ cells among CD3^+^CD8^+^ cells from TDLNs. I) Flow cytometry analyses of OVA‐tetramer binding CD8^+^ cells from TDLNs. One‐way ANOVA followed by Tukey's posthoc test was calculated using GraphPad PRISM (^*^ *p* < 0.05, ^**^ *p* < 0.01, ^***^ *p* < 0.001, ^****^ *p* < 0.0001). Data are presented as the mean±SD. TFM, tumor free mice.

The most important aspect of a cancer vaccine is its ability to prime the immune system in a tumor‐specific manner and prevent the occurrence or recurrence of cancer. We explored the potential of AI‐TEVs as a prophylactic vaccine by challenging E.G7‐OVA tumor cells with weekly subcutaneous injections of TEVs (**Figure** **4**A). AI‐TEV vaccination effectively delayed tumor growth compared to the other groups, with more than half of the experimental animals maintaining a tumor‐free state for almost 3 weeks (Figure 4B). Mice injected with C‐TEVs also showed delayed progression of tumor growth, which was expected due to the high immunogenicity of E.G7‐OVA cells and the fact that C‐TEVs are already known to carry tumor antigens. Therefore, a somewhat limited prophylactic effect was predicted for C‐TEVs; however, we validated that this effect was amplified in a tumor‐specific manner in AI‐TEVs. This prophylactic effect was also demonstrated using MOC2 TEVs (Figure S5A,B, Supporting Information).

To validate whether AI‐TEVs induce tumor‐specific immunity, we extracted the spleens and tumor‐draining lymph nodes (TDLNs) from mice vaccinated with E.G7‐OVA TEVs (Figure 4C). The immune cell portions of the spleen were assessed via flow cytometry which revealed a significant increase in CD8^+^ T cells in the AI‐TEV group. Among these T cells, CD107a^+^ was used to identify cytotoxic CD8^+^ T cells, which also demonstrated a significant increase in the AI‐TEV group (Figure 4D). Interestingly, while there were no substantial changes in the CD4^+^ T cell or NK cell population in the spleen, there was an increase of CD107a^+^ in CD4^+^ T cells in the AI‐TEV group (Figure S5C,D, Supporting Information). Separately, the splenocytes were pulsed with two types of irradiated cancer cells (E.G7‐OVA or B16F10 as a control) ex vivo. Flow cytometric analysis of the splenocytes to determine the percentage of activated effector CD8^+^ T cells (Figure S6A, Supporting Information) revealed a much higher proportion of IFN‐γ^+^ CD44^+^ CD8^+^ T and TNF‐α^+^ CD44^+^ CD8^+^ T cells in splenocytes of mice vaccinated with AI‐TEVs than that in mice vaccinated with C‐TEVs and PBS. Importantly, splenocytes pulsed with B16F10 cells, rather than their respective cancer cells, showed no notable differences, suggesting a tumor‐specific response of CD8^+^ T cells (Figure 4E; Figure S5E,F, Supporting Information) and the ability of AI‐TEVs to educate splenocytes. The same assay to detect tumor‐specific response of CD4^+^ T cells and NKT cells showed no significant differences between all experimental groups (Figure S5H,I, Supporting Information). To further verify the tumor‐specific immune response, splenocytes from vaccinated mice were labeled with carboxyfluorescein diacetate‐succinimidyl ester (CFSE) and incubated with irradiated E.G7‐OVA cells for 72 h. Flow cytometric analysis of CFSE‐positive CD8^+^ T cells to determine their proliferation revealed a highly significant dilution of CFSE in AI‐TEV group compared to that in other groups, suggesting a compelling tumor‐specific reaction (Figure 4F). Moreover, a significant increase in mature DCs (CD40^+^ CD11c^+^ and CD86^+^ CD11c^+^) and antigen‐experienced CD8^+^ T cells (CD44^+^ CD8^+^ T cells) was observed in TDLN cells, suggesting an enhancement in the ability of DCs to prime CD8^+^ T cells in the lymph nodes (Figure 4G,H; Figures S5G and 7A, Supporting Information). To further confirm this theory, we stained TDLN cells with MHC tetramers to detect SIINFEKL‐specific T cell populations (Figure 4I). This tetramer assay revealed a significant difference in the OVA‐tetramer^+^ CD8^+^ T cell population, which showed an increase in the percentage of tumor antigen‐specific CD8^+^ T cells in AI‐TEV‐injected mice. These results validate the potential of AI‐TEVs as prophylactic vaccines that can induce tumor‐specific immunity.

### 2.5 AI‐TEVs Elicit Long‐Term Memory, Effectively Preventing Tumor Recurrence

**Figure 5:**
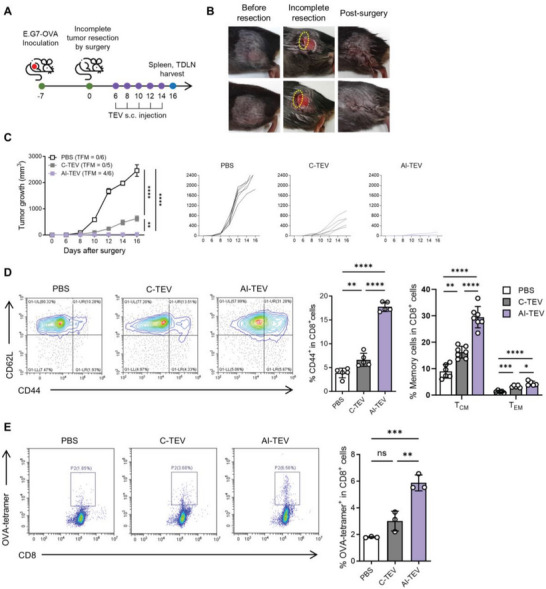
AI‐TEVs can prevent tumor recurrence by activating long‐term memory and tumor‐specific immunity. A) Incomplete resection model of E.G7‐OVA tumors to observe the effects of vaccination on tumor recurrence. Spleens and TDLNs were harvested by the end (Day 16). B) Representative photographs of incomplete resection of tumors on mice. C) Average tumor growth curves (n = 5‐6 per groups) and individual tumor growth curves. D) Splenocytes were analyzed through flow cytometry to assess the percentage of CD44^+^CD8^+^ T cells, CD44^hi^CD62L^hi^ T cells, and CD44^hi^CD62L^lo^ T cells. E) Flow cytometry analyses of OVA‐tetramer binding CD8^+^ cells from TDLNs. One‐way ANOVA followed by Tukey's posthoc test was calculated using GraphPad PRISM (^*^ *p* < 0.05, ^**^ *p* < 0.01, ^***^ *p* < 0.001, ^****^ *p* < 0.0001). Data are presented as the mean±SD.

Although traditional vaccines are meant to completely prevent the occurrence of a target disease, cancers are limited because not every occurrence is preventable or predictable. Few cancers have specific causes or triggers that can be avoided. Therefore, we aimed to develop a cancer vaccine that could act as an adjuvant therapy to be used after the primary eradication of tumors, designed to target DCs, and educate the immune system to prevent the recurrence or progression of residual cancer. In case the initial cancer treatment (surgical resection or chemotherapy) fails to eradicate the cancer, these vaccines should be able to slow down or prevent further cancer growth. We explored the potential of AI‐TEVs as a post‐surgical immunotherapy to prevent cancer recurrence by establishing a tumor recurrence mouse model using E.G7‐OVA cells (**Figure** **5**A). After incomplete resection of the primary tumor (≈10% mass was retained) (Figure 5B), TEV therapy was initiated 6 days post‐surgery (once every 2 days, for a total of five injections) and recurrent growth of tumor was monitored. AI‐TEV injection significantly prevented tumor recurrence, with four out of six mice showing complete tumor regression by the end of the experimental period (day 16) (Figure 5C). Analysis of the spleen indicated a much greater proportion of CD44^+^ CD8^+^ T cells in the AI‐TEV group, and a considerable increase in CD44^hi^ CD62^hi^ CD8^+^ T cells (central memory T cells, T_CM_) along with a lesser but still significant increase in CD44^hi^ CD62L^lo^ CD8^+^ T cells (effector memory T cells, T_EM_) (Figure 5D; Figure S8A, Supporting Information). Furthermore, a tetramer assay of TDLN cells revealed a significant increase in the proportion of OVA‐tetramer^+^ CD8^+^ T cells, validating that long‐term memory induced by AI‐TEVs is tumor antigen‐specific (Figure 5E). Taken together, these results suggest that AI‐TEVs can induce a powerful boost in tumor‐specific memory from an immune standpoint, indicating their potential as adjuvant therapy to prevent the recurrence of residual cancer.

### 2.6 AI‐TEVs Can Be Used as a Personalized Cancer Vaccine Platform

**Figure 6:**
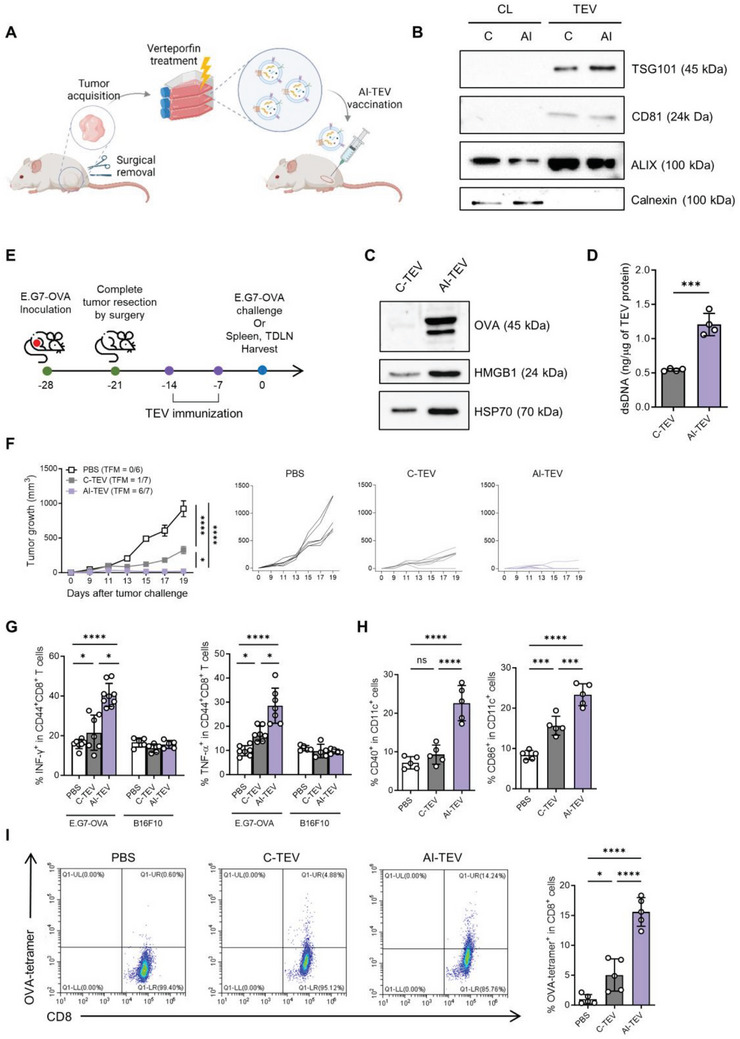
AI‐TEVs effectively prevent tumor growth and induce tumor‐specific immunity in a personalized cancer vaccine model. A) Personalized cancer vaccine model scheme. Mice were inoculated with E.G7‐OVA tumors, after which the tumors were completely resected by surgery, dissociated, and cultured. The same mice were vaccinated with TEVs isolated from these cells twice, then challenged with the same tumor cells. B) Western blotting of cell lysates and TEVs detecting EV markers TSG101, CD81, and Alix, along with a negative marker, Calnexin. C) Western blotting of TEVs detecting OVA and DAMPs (HMGB1, HSP70). D) dsDNA loaded onto TEVs were quantified using a QuantiFluor dsDNA system. E–I) Mice were injected with TEVs isolated from their completely resected tumors, once weekly, a total of two times. They were then either challenged with these tumor cells or harvested for their spleen and TDLN. E) Vaccination schedule of mice inoculated with E.G7‐OVA cells. F) Vaccinated mice were challenged with E.G7‐OVA cells and their tumor size were monitored. Average tumor growth curves (*n* = 6–7 per group) and individual tumor growth curves for each group. G) Vaccinated mice were harvested of their spleen. The splenocytes were stimulated with UV‐irradiated cancer cells for 5 h and analyzed with flow cytometry to identify the percentage of IFN‐γ^+^ and TNF‐α^+^ cells among CD44^+^CD8^+^ cells. (H) Vaccinated mice were harvested of their TDLNs. Flow cytometry analyses of the percentage of CD40^+^ and CD86^+^ cells among CD11c^+^ cells from TDLNs. I) Flow cytometry analyses of OVA‐tetramer binding CD8^+^ cells from TDLNs. One‐way ANOVA followed by Tukey's posthoc test was calculated using GraphPad PRISM (^*^ *p* < 0.05, ^**^ *p* < 0.01, ^***^ *p* < 0.001, ^****^ *p* < 0.0001). Data are presented as the mean±SD.

The importance of personalized cancer vaccines is evident owing to the fact that cancer cells are known for their aberrant and mutative behavior, highlighting the need to trigger personalized immune responses against neoantigens.^[^ ^32^ ^]^ The application of autologous tumor cells directly derived from individual patients to isolate AI‐TEVs as personalized vaccines is a promising therapeutic approach, which we aimed to prove using a personalized cancer vaccine model. Tumors were induced in mice by E.G7‐OVA tumor cell inoculation, completely resected by after 7 days and suspended in media for culture. TEVs were isolated from the cultured tumor cells and used for vaccination in the same mice, who were then challenged with the same cells or euthanized for analysis (**Figure** **6**A). Immunoblotting of these TEVs revealed the presence of EV markers (Figure 6B) and an increase in OVA protein and DAMPs in AI‐TEVs compared to C‐TEVs (Figure 6C). Quantitation of the dsDNA cargo revealed a greater increase in dsDNA in AI‐TEVs than that in C‐TEVs (Figure 6D). These data suggest that personalized AI‐TEVs carry high levels of tumor antigens and adjuvants, making them suitable for vaccination. The prophylactic effect of TEV vaccination in a personalized cancer vaccine model was demonstrated by rechallenging vaccinated mice with their previously challenged E.G7‐OVA cells (Figure 6E). AI‐TEVs showed a dramatic outcome, resulting in tumor‐free state in all but one mice after almost 3 weeks (Figure 6F). Further analysis of the spleen and TDLNs of these vaccinated mice validated a significant *ex vivo* activation of tumor‐pulsed splenocytes, determined by an increased proportion of IFN‐γ^+^ and TNF‐α^+^ CD44^+^ CD8^+^ T cells (Figure 6G). Analysis of TDLN cells revealed an increase in the number of mature DCs (CD40^+^ CD11c^+^ and CD86^+^ CD11c^+^) and OVA‐tetramer^+^ CD8^+^ T cells (Figure 6H,I). These data suggest that personalized AI‐TEVs can elicit robust and tumor‐specific immune responses, potentially acting as tumor vaccines designed at the individual level.

## 3 Discussion

In this study, we have designed attenuated and immunogenically potentiated TEVs (AI‐TEVs) using the mechanism of verteporfin action. Verteporfin‐treated tumor cells secreted TEVs with attenuated pro‐tumorigenic features and enhanced immunogenic properties. Moreover, AI‐TEVs contained large quantities of dsDNA, which is a potent adjuvant that can elicit effective immune responses against tumors. We have validated that AI‐TEV vaccines can mediate robust tumor‐specific immune responses and induce long‐lasting memory responses by assessing their ability to prevent tumor growth in prophylactic and recurrence models. This provides insights into the clinical relevance of these cancer vaccines. Furthermore, by developing a personalized cancer vaccine model, we have demonstrated the potential of AI‐TEVs to be used as a modality for personalized cancer vaccines that can be designed for patient‐specific cancers.

With the discovery of the immune system's ability to retain the memory of external antigens, vaccination has become an essential part of immune studies. Cancer is no exception, and cancer vaccination is an actively studied field of cancer immunotherapy, albeit with limited clinical outcomes.^[^ ^33^, ^34^ ^]^ Among these, personalized cancer vaccination offers a unique approach by tailoring treatment to the distinct and ever‐evolving nature of each patient's cancer.^[^ ^1^, ^32^ ^]^ The selection of appropriate tumor antigens, delivery systems, and sufficient adjuvants to overcome the immunosuppressive tumor environment are the challenges in the development of personalized cancer vaccines.^[^ ^2^, ^35^ ^]^

Numerous studies have determined the antitumor efficacy of TEVs, which are tumor cell‐derived bilipid‐layer vesicles primarily known for their role in tumor progression and immunosuppression of the tumor microenvironment.^[^ ^9^ ^]^ However, the most important aspect of TEVs is their ability to shuttle tumor antigens throughout the body, making them an indisputably valuable tool for stimulating the immune system.^[^ ^9^, ^11^ ^]^ Although numerous attempts have been made to manipulate TEVs for cancer immunotherapy, most of these modifications aimed to increase the antitumor efficacy of TEVs through complicated methods, such as genetic or surface modification or loading TEVs with cytotoxic drugs.^[^ ^36^, ^37^, ^38^ ^]^ While these methods may compensate for the lack of immunogenicity of the original TEVs, they do not enhance the innate tumor‐specific antigenicity and immune‐stimulating ability of TEVs, nor do they address the important issue of debilitating their tumor‐promoting characteristics. In the present study, we provide a method to develop TEV cancer vaccine using verteporfin that utilizes autologous TEVs as a source of antigens and adjuvants to boost tumor‐specific immunity while silencing their malignant properties. These AI‐TEVs developed in the present study are an ideal mode of cancer vaccination.

The AI‐TEVs carried high amounts of tumor antigens and adjuvants, such as DAMPs and dsDNA, while their tumorigenic characteristics were effectively attenuated, as confirmed at the proteomic and functional levels. As a result, AI‐TEVs not only induced the maturation and activation of DCs in vitro but were also highly capable as therapeutic and prophylactic vaccines in vivo. We further confirmed that AI‐TEVs could elicit tumor‐specific and long‐term immunity, validating their potential as a post‐surgical immunotherapy in a tumor recurrence model. Finally, we demonstrated the potential of AI‐TEVs as a personalized vaccine by establishing a murine personalized cancer vaccination model that emulates clinical conditions. There have been significant advances in efforts to identify tumor‐specific somatic mutations and their subsequent neoantigens using complex processes, such as whole‐exosome sequencing and various computational algorithms. However, these methods are too complicated for general clinical use and usually focus on MHC I epitopes and not on inducing cytotoxic CD8^+^ T cell responses.^[^ ^32^, ^39^ ^]^ The present study particularly harnessed tumor‐derived antigens loaded onto TEVs to ensure their neoantigen content and ensure their safe delivery to elicit a robust response in CD8^+^ T cells and thereby develop an effective personalized cancer vaccine.

E.G7‐OVA lymphoma cells are mainly used in the present study because OVA expression makes them an appropriate cell line for determining whether AI‐TEVs could induce tumor‐specific immunity. However, this could also be a limitation of our research as OVA overexpression could make E.G7‐OVA a highly immunogenic cancer type. We compensated for this drawback by including MOC2 and 4T1 cells, both of which are poorly immunogenic and highly aggressive. Further experiments on the use of AI‐TEVs targeting aggressive tumors as a treatment model, especially with immune checkpoint blockades, may induce a much more reliable and potent tumor‐suppressing effect, as cancer vaccines are well known to be used in combination with other immunotherapies.^[^ ^40^ ^]^

In summary, we have developed an improved form of TEVs as a cancer vaccine that can elicit robust tumor‐specific responses and effectively suppress cancer growth in prophylactic as well as post‐surgical settings. More importantly, AI‐TEVs can be utilized as personalized cancer vaccine platforms, making them an encouraging new approach for customizable cancer immunotherapy.

## 4 Experimental Section

### Cell Culture

E.G7‐OVA murine thymoma cells and 4T1 murine breast cancer cells were purchased from ATCC (Manassas, VA, USA) and cultured in Roswell Park Memorial Institute Medium (RPMI 1640; SH30255.01; Hyclone, Logan, UT, USA) supplemented with 10% fetal bovine serum (FBS; 12483‐020; Gibco, Billings, Montana, USA) and 1% antibiotic–antimycotic (15240‐062; Gibco). MOC2 cells were also purchased from ATCC and cultured in Iscove's modified Dulbecco's medium (SH3022902; Hyclone) supplemented with 5% FBS, 1% penicillin–streptomycin solution (SV30010; Hyclone), 31.3% Ham's nutrient mixture F12 (SH30026.01; Hyclone), 5 µg mL^−1^ insulin (16634‐50 mg; Sigma–Aldrich, Burlington, MA, USA), 0.04 µg mL^−1^ hydrocortisone (H0135‐1 mg; Sigma–Aldrich), and 0.005 µg mL^−1^ recombinant human epidermal growth factor (EGF; C029; Novoprotein, Suzhou, China). All cells were incubated at 37 °C and 5% CO_2_.

### TEV Preparation and Isolation

When the cancer cells reached 80–90% confluence, they were incubated in serum‐free media containing verteporfin (20 ng mL^−1^ for E.G7‐OVA cells and 200 ng mL^−1^ for 4T1 and MOC2 cells) for 24 h. Verteporfin was purchased from Sigma–Aldrich (SML0534). Cell supernatants underwent a series of centrifugation steps; 300 × *g* for 10 min, 2000 × *g* for 10 min, and 10 000 × *g* for 30 min to eliminate microvesicles and cellular debris. Subsequently, the resultant supernatants were filtered through a 0.22 µm pore filter (431 118; Corning, Corning, NY, USA), dialyzed with PBS via tangential flow filtration (KrosFlo KR2i, Repligen, Boston, MA, USA) using a MIDIKROS filter (41.5 cm 300 K MPES 0.5 mm; Repligen), and then centrifuged at 150 000 × *g* for 2 h. The pellets were reconstituted in PBS containing proteinase inhibitor cocktail (PIC; 11 697 498 001; Roche, Basel, Switzerland) and preserved at 4 °C.

### Nanoparticle Tracking Analysis

The size and number of TEVs were measured using ZetaView (Particle Metrix, Meerbusch, Germany) and the corresponding software (ZetaView 8.02.28). After calibrating the ZetaView system with polystyrene beads (3090A, ThermoFisher Scientific, Waltham, MA, USA), exosomes were diluted and loaded for analysis to obtain size distribution and particle number. Samples were analyzed at 11 different positions throughout the chamber. Experiments were repeated at least four times and the mean value is presented in the figure.

### Cryogenic Transmission Electron Microscopy

TEVs were visualized through the use of cryogenic transmission electron microscopy (Tecnai Systems, Philips, Holland). The purified TEVs were deposited onto a thin carbon film on copper grids and then cryogenically preserved with the use of a Vitrobot (FP5350; FEI).

### Immunoblotting

The samples’ protein concentrations were quantified using a DC Protein assay kit (5 000 111; Bio‐Rad, Hercules, CA, USA). The samples were lysed using a radioimmunoprecipitation buffer (9806S; Cell Signaling Technology, Danvers, MA, USA) and subjected to SDS‐PAGE. Samples were loaded onto a sodium dodecyl sulfate‐polyacrylamide gel, separated by gel electrophoresis, and transferred onto a nitrocellulose membrane. The membranes were blocked with 5% skim milk and then incubated with primary antibodies against Tsg101 (sc‐22774; Santa Cruz, Dallas, TX, USA), YAP (sc‐99010), CD81 (sc‐166029), ALIX (sc‐53540), calnexin (ab22595; Abcam, Cambridge, United Kingdom), OVA (0220‐1682; Bio‐Rad), HMGB1 (ab18256; Abcam), HSP70 (ab181606; Abcam), or β‐actin (4967S; Cell Signaling Technology) overnight at 4 °C. After washing five times (5 min each) with Tris‐buffered saline containing 0.05% Tween‐20 (TBS/T), the membranes were incubated with anti‐mouse peroxidase (1:3000; A4416; Sigma–Aldrich) or anti‐rabbit peroxidase (1:3000; A0545) secondary antibodies for 1 h at 20 to 25 °C. Membranes were then incubated with ECL substrate (1 705 061; Bio‐Rad) and visualized using a ChemiDoc Touch Imaging System (Bio‐Rad).

Each immunoblotting experiments were performed at least three times and the representative western blots are presented in the figures.

### Cell Viability Assay

Cells were seeded in a 96‐well plate (30 096; SPL Life Sciences, Pochon, South Korea) (5000 cells/well) and treated with various doses of verteporfin in serum‐free media for 24 h. Cell viability was measured using the Cell counting kit‐8 (CCK‐8) assay (CK04; Dojindo, Kumamoto, Japan), and absorbance was measured at 450 nm using a microplate reader (SpectraMAX 340; Molecular Devices, San Jose, CA, USA).

### Calreticulin Analysis

Cells were seeded onto a six‐well plate (30 006; SPL Life Sciences) (5 × 10^5^ cells per well) and treated with verteporfin in serum‐free medium for 3 h. Cells were fixed with 0.25% paraformaldehyde for 5 min at 4 °C and then blocked with 3% bovine serum albumin for 15 min at 4 °C. Calreticulin antibody (ab2907; Abcam) was added to the samples and incubated at 4 °C for 45 min. After washing with PBS, Alexa Fluor 488‐conjugated secondary antibody (711‐545‐152; Jackson ImmunoResearch, West Grove, PA, USA) was added to the sample and incubated for 30 min at 4 °C. Samples were washed using Dulbecco's Phosphate‐Buffered Saline (DPBS; LB001‐02; Welgene, Taipei, China) and stained with propidium iodide (PI; P4864; Sigma–Aldrich) at 44 °C for 15 min to check cell viability. Calreticulin expression on the cell membrane was assessed using flow cytometry gating of the PI‐negative cells.

### Evaluation of the Effect of TEVs on DCs

Bone marrow cells were collected from 6–8 week‐old C57BL/6 mice (Orient Bio, Gwangju, South Korea) and cultured in RPMI 1640 medium (LM011‐01; Welgene) supplemented with 10% FBS (12483‐020; Gibco) and 1% antibiotic–antimycotic solution (15240‐062; Gibco). The cells were differentiated using 20 ng mL^−1^ recombinant murine granulocyte‐macrophage colony‐stimulating factor (GM‐CSF; 315‐03; PeproTech, Cranbury, NJ, USA), 20 ng mL^−1^ recombinant murine IL‐4 (214‐14; PeproTech), and 0.1% β‐mercaptoethanol (21 985 023; Gibco) for 7 days.

To verify DC maturation and the activation of the cGAS‐STING pathway, the differentiated DCs were co‐cultured with 10 µg mL^−1^ TEVs on day 8 for 24 h and then analyzed via flow cytometry using APC‐conjugated anti‐CD11c (117 310; BioLegend, San Diego, CA, USA) and maturation markers PE‐conjugated anti‐CD40 (124 610; BioLegend), anti‐CD86 (105 008; BioLegend), anti‐CD80 (104 707; BioLegend), and FITC‐conjugated anti‐I‐A/I‐E (107 606; BioLegend). Anti‐H‐2Kb antibody bound to SIINFEKL (141 603; BioLegend) was used to analyze cross‐presentation. The cells were fixed and permeabilized using the CytoFix/CytoPerm kit (BD 554 714; BD Biosciences, Franklin Lakes, NJ, USA) and then subjected to intracellular staining with anti‐phospho‐TBK1/NAK (13 498; Cell Signaling Technology) and anti‐phospho‐IRF3 (83611S; Cell Signaling Technology) antibodies. The supernatant of TEV‐treated DCs was collected, and the concentration of IFN‐γ was detected with a mouse IFN‐β Quantikine ELISA kit (MIFNB0; R&D Systems, NE Minneapolis, MN, USA).

### dsDNA Quantification

TEVs were incubated with Triton‐X and Proteinase K for 30 min at 50 °C. Then, dsDNA was quantified using the QuantiFluor dsDNA System (E2671; Promega, Madison, WI, USA) and a GloMax Discover microplate reader (Promega).

### Transwell Assay

For the migration assay, 6.5 mm Transwell (CLS3422; Corning) with 8.0 µm Pore Polyester Membrane inserted in an empty 24‐well plate was used. For the invasion assay, the membrane was coated with 0.5 mg mL^−1^ Matrigel that was dried for 24 h at 37 °C before use. Cancer cells were seeded in the apical chamber with 1% FBS, and the basal chamber was filled with serum‐free medium. Cells were co‐cultured with TEVs (10 µg mL^−1^) in the apical chamber. After 24 h, the Transwell membrane was washed twice with PBS, fixed with 4% paraformaldehyde, washed again with PBS, and stained with 0.1% crystal violet. After washing with PBS, the membranes were observed under an optical microscope. Stained cells were counted using ImageJ software (National Institutes of Health, Laboratory for Optical and Computational Instrumentation).

### Proteomics

The concentration of TEVs was quantified using a Pierce BCA Protein Assay Kit (23 225; Thermo Fisher Scientific). Following digestion, the samples were eluted with a solution consisting of 80% acetonitrile and 0.1% formic acid (Honeywell, Charlotte, NC, USA) in water, following the manufacturer's guidelines. Subsequently, the samples reconstituted in a solution of 0.1% formic acid in water and analyzed using a Q‐Exactive Orbitrap hybrid mass spectrometer (Thermo Fisher Scientific) in combination with an Ultimate 3000 system (Thermo Fisher Scientific). The raw Thermo MS/MS data files for each analysis were subjected to a search using Proteome DiscovererTM software (version 2.5), and the Musculus database was sourced from UniProt (https://www.uniprot.org/). The relevant proteomics data ratios were then assessed using ExDEGA version 3.0.1 (ebiogen, Seoul, Korea).

### Animals

Male C57BL/6 (6–7 weeks old) and female BALB/c (6–7 weeks old) mice were purchased from Orient Bio and housed under environmentally controlled conditions (23 ± 2 °C, relative humidity of 55 ± 10%, and a 12‐h light–dark cycle) with ad libitum access to food and water at a specific‐pathogen free animal facility in the Korea Institute of Science and Technology (KIST; Seoul, Republic of Korea). The protocols for the animal experiments were approved by the Association for Assessment and Accreditation of Laboratory Animal Care, KIST (approval no. KIST‐IACUC‐2020‐095).

### In Vivo Tumor Models

A therapeutic vaccination model was established by subcutaneously inoculating 5 × 10^5^ E.G7‐OVA tumor cells into the left flank of C57BL/6 mice. Five days after inoculation, 20 µg of TEVs were subcutaneously injected adjacent to the tumor inoculation site once every 2 days, for a total of five times.

The prophylactic vaccination model was established by subcutaneously injecting 40 µg of TEVs into the right flank of C57BL/6 or BALB/c mice once a week, a total of two times. One week after the last vaccination, ≈5 × 10^5^ tumor cells were subcutaneously inoculated into the left flank of the mice and observed every 2 days. Tumor size was determined by calculating the area using the formula (width)^2^ × (length) / 2, and mice with tumors >2000 mm^3^ were euthanized and their TDLNs and spleens were harvested for analysis.

A tumor recurrence model was established by subcutaneously inoculating ≈5 × 10^5^ E.G7‐OVA tumor cells into the left flank of C57BL/6 mice. Following a 7‐day period, when the tumor had grown to ≈100 mm^3^ in size, it was incompletely resected, leaving ≈10% of the tumor mass on the tumor bed. Mice with tumors that did not reach the appropriate size were excluded. Six days after resection, 20 µg of TEVs were subcutaneously injected adjacent to the tumor inoculation site once every 2 days, for a total of five times. The mice were euthanized and their TDLNs and spleens were harvested for analysis 16 days after resection.

A personalized cancer vaccine model was established by subcutaneously inoculating ≈1 × 10^6^ E.G7‐OVA tumor cells into the right flank of C57BL/6 mice. Following a 7‐day period, when the tumor had grown to ≈300 mm^3^ in size, the tumors were completely resected and dissociated using a tumor dissociation kit (130‐096‐730; Miltenyi Biotec, Bergisch‐Gladbach, Germany) and a GentleMACS dissociator (Miltenyi Biotec). Dead cells were eliminated using a dead cell removal kit (130‐090‐101; Miltenyi Biotec) and the remaining live cells were suspended in serum‐containing RPMI. After 24 h, the cells that adhered to the culture flask were discarded, and the suspended cells were re‐suspended in RPMI for culture. The TEVs isolated from these cells were subcutaneously injected into the left flank of the same mouse once a week, a total of two times. One week after the last vaccination, mice were either challenged with cultured cancer cells to monitor tumor growth or euthanized, and their spleens and TDLNs were harvested.

### Ex Vivo Analysis

To ascertain T cell activation, spleen was isolated from vaccinated mice, disassociated using a GentleMACS dissociator (Miltenyi Biotec), and seeded into a 96‐well round‐bottom plate at 5 × 10^5^ cells/well. Cancer cells were irradiated for 30 min under UV light and added to the seeded splenocytes at 1 × 10^5^ cells/well. After 1 h, protein transport inhibitors BD GolgiStopTM (containing Monensin; BD 554 724; BD Biosciences) and BD GolgiPlugTM (containing Brefeldin A; BD 555 029; BD Biosciences) were added and the cells were incubated for another 4 h. Then, the cells were collected, fixed, and permeabilized using a CytoFix/CytoPerm kit (BD 554 714; BD Biosciences). Cells were blocked with anti‐mouse CD16/CD32 (553 142; BD Biosciences) and intracellularly stained with the following antibodies purchased from BioLegend: anti‐CD45.2 (109 837), anti‐CD3 (100 221), anti‐CD8a (100 705), anti‐CD44 (103 008), anti‐IFN‐γ (505 824), and anti‐TNF‐α (506 308).

To ascertain T cell proliferation, splenocytes were labeled with CFSE using the CellTraceTM CFSE Cell Proliferation Kit (C34554; Thermo Fisher Scientific). The labeled splenocytes were seeded into a 96‐well round‐bottom plate at 5 × 10^5^ cells/well. Approximately 1 × 10^5^ irradiated cancer cells were stimulated with IL‐2 (100 ng mL^−1^) for 72 h. Then, cells were collected and stained with the following antibodies purchased from BioLegend: anti‐CD45.2 (109 830), anti‐CD3 (100 227), and anti‐CD8a (100 712).

### Flow Cytometric Analysis

The TDLNs and spleens harvested from the experimental mice were gently mashed by hand or using a GentleMACS dissociator (Miltenyi Biotec). The disassociated cells were filtered through a 40 µm strainer, red blood cells were lysed using red blood cell lysis buffer (BioLegend), and the cells were FC‐blocked with anti‐mouse CD16/CD32 (553 142; BD Biosciences).

The following antibodies were used for flow cytometric analysis that were purchased from BioLegend: anti‐CD11c (117 310), anti‐CD40 (124 610), anti‐CD86 (105 008), anti‐CD45.2 (109 830), anti‐CD3 (100 222), anti‐CD8a (100 706), anti‐CD44 (103 023), anti‐CD62L (104 412), anti‐CD4 (100 414), anti‐NK1.1 (156 508, 108 738) and anti‐CD107a (121 612). Tetramer/BV421–H‐2 Kb OVA (SIINFEKL) (TB‐5001‐4) was purchased from MBL (Sunnyvale, CA, USA). Flow cytometry data were analyzed using FlowJo (v10) software (TreeStar, San Francisco, CA, USA) and CytExpert (v2.5) software (Beckman Coulter Life Sciences, Brea, CA, USA).

### Statistical Analysis

All data are presented as mean ± standard deviation (SD) or standard error of the mean (SEM) for control and experimental samples. Multigroup comparisons were performed using ANOVA followed by Tukey's post‐hoc test. Statistical significance set at using 95% (p < 0.05), 99% (p < 0.01), 99.9% (p < 0.001), and 99.99 (p<0.0001) confidence intervals. GraphPad Prism 9.5.0 (GraphPad Software, San Diego, CA, USA) was used for the statistical analyses.

## Conflict of Interest

The authors declare no conflict of interest.

## Author Contributions

J.H. and S.K. contributed equally to this work. J.H and S.K designed the study and performed the experiments. Y.H.H., S.A.K., Y.L., J.K., S.C., and J.W. assisted with the animal experiments. J. H and S. K wrote the manuscript, and C.J., M.K., G.‐H.N., I.‐S.K. revised the manuscript. All authors discussed the results of the experiments and contents of the manuscript.

## Supporting information

Supporting Information

## Data Availability

The data that support the findings of this study are available in the supplementary material of this article.
