# Supplementary material for: Novel Personalized Cancer Vaccine Using Tumor Extracellular Vesicles with Attenuated Tumorigenicity and Enhanced Immunogenicity
Source: Adv Sci (Weinh). 2024 Apr 26;11(25):2308662. doi: 10.1002/advs.202308662 (PMC11220679; doi:10.1002/advs.202308662)
Supplement: Supplementary file 1 — Supporting Information [file ADVS-11-2308662-s001.pdf]

ADVANCED SCIENCE
Open Access

# Supporting Information

for *Adv. Sci.*, DOI 10.1002/advs.202308662

Novel Personalized Cancer Vaccine Using Tumor Extracellular Vesicles with Attenuated Tumorigenicity and Enhanced Immunogenicity

*Jihoon Han, Seohyun Kim, Yeong Ha Hwang, Seong A Kim, Yeji Lee, Jihong Kim, Seongeon Cho, Jiwan Woo, Cherlhyun Jeong, Minsu Kwon, Gi-Hoon Nam and In-San Kim**

Supporting Information

**Novel Personalized Cancer Vaccine Using Tumor Extracellular Vesicles with Attenuated Tumorigenicity and Enhanced Immunogenicity**

*Jihoon Han, Seohyun Kim, Yeong Ha Hwang, Seong A Kim, Yeji Lee, Jihong Kim, Seongeon Cho, Jiwan Woo, Cherlhyun Jeong, Minsu Kwon, Gi-Hoon Nam, In-San Kim**

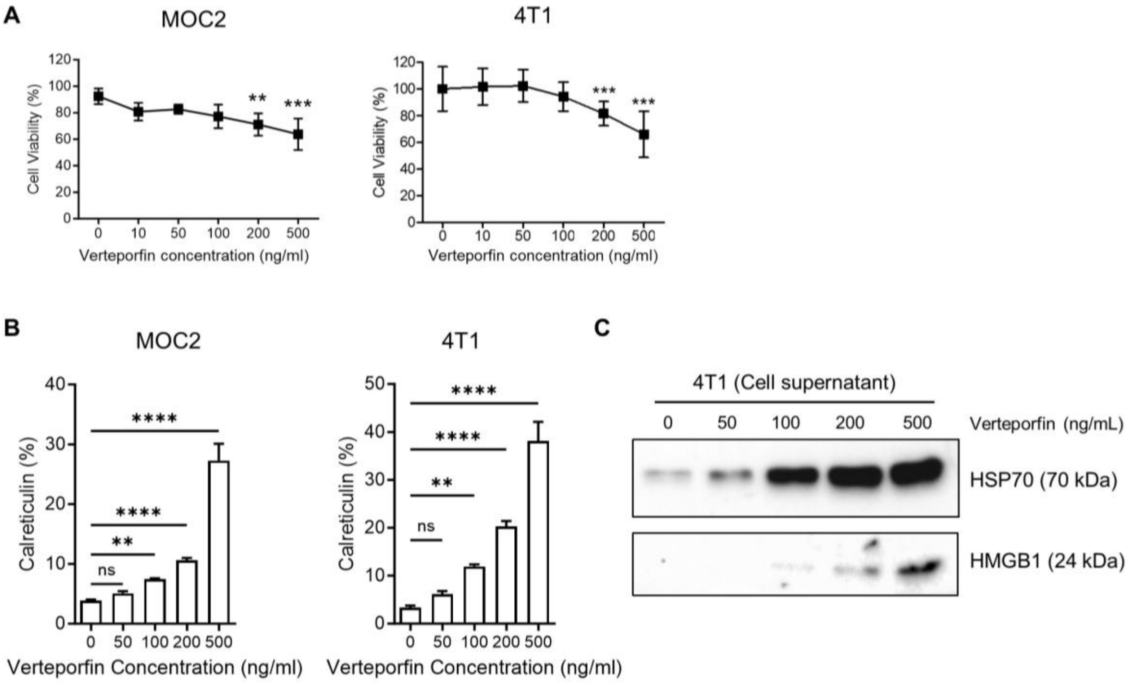


**Figure S1.** Verteporfin induces ICD in MOC2 and 4T1 cells. A) MOC2, and 4T1 cells were cultured in with different concentrations of verteporfin for 24 h. Total viable cells were measured with a CCK-8 assay. B) Flow cytometry analysis of calreticulin expression on the surface of cell membrane-gated PI- intact cells. C) Western blotting of HSP70 and HMGB1 released in conditioned media 24 h after verteporfin treatment.
One-way ANOVA followed by Tukey's posthoc test was calculated using GraphPad PRISM (*p<0.05, **p<0.01, ***p<0.001, ****p<0.0001). Data are presented as the mean±SD.

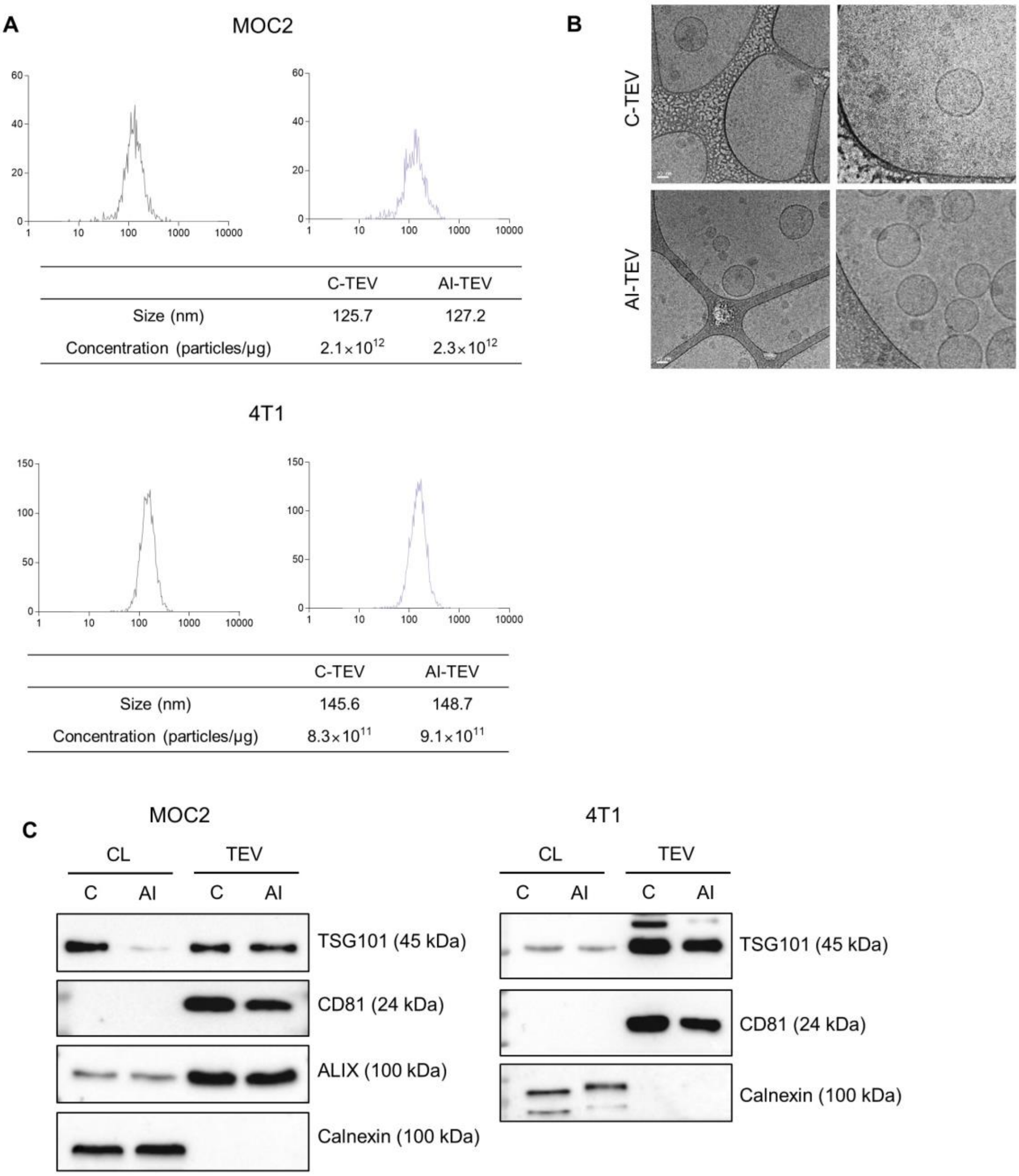


**Figure S2.** Characterization of C-TEVs and AI-TEVs in MOC2 and 4T1 cancer cells. A) Size distribution and particle number of TEVs, as assessed using nanoparticle tracking analysis (NTA). B) Cryo-TEM image of C-TEVs and AI-TEVs secreted by 4T1 cells (scale bar: 50nm). C) Western blotting of cell lysates and TEVs detecting EV markers TSG101, CD81 and Alix, along with a negative marker, calnexin.
CL, Cell lysate

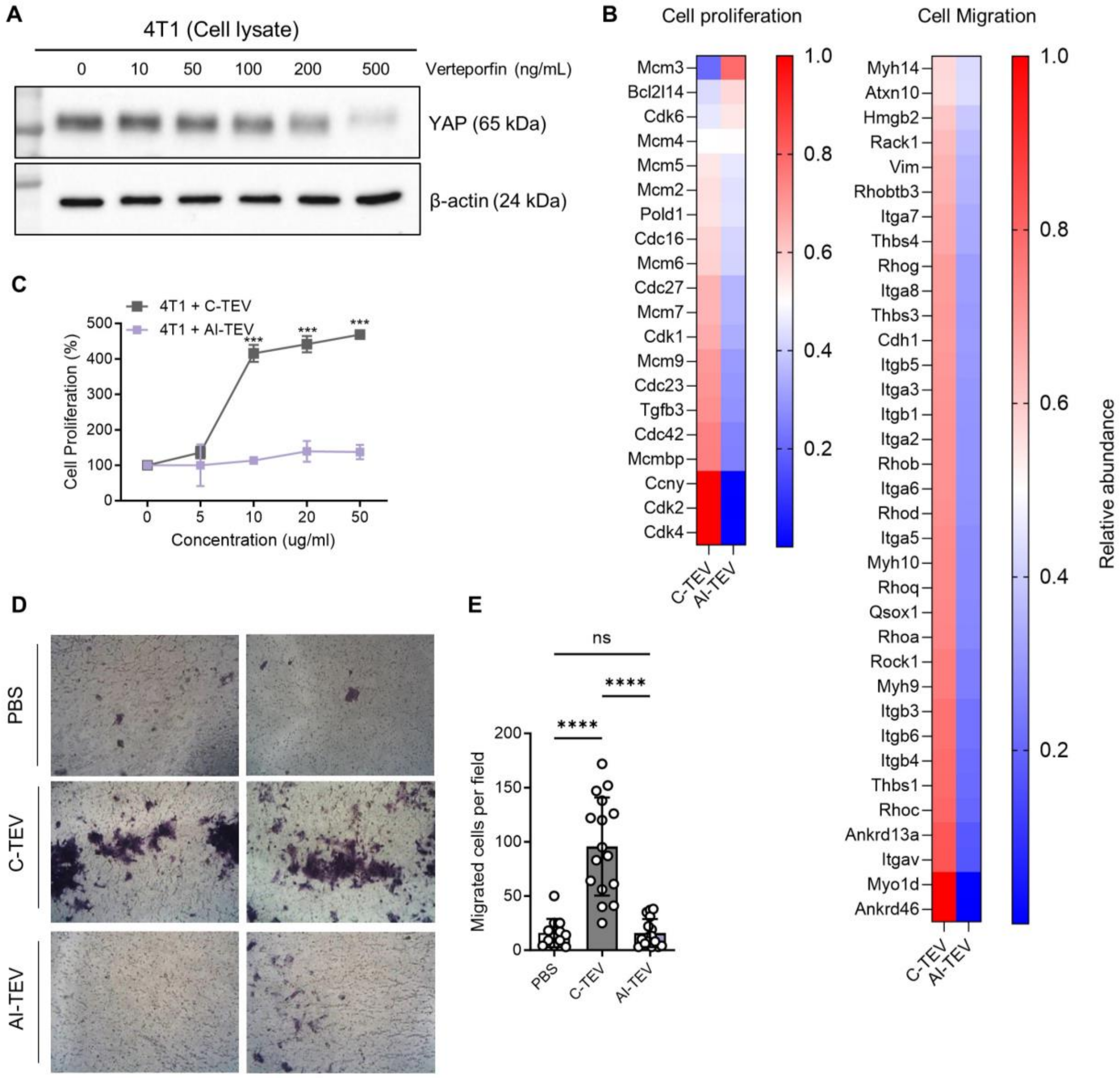


**Figure S3.** Verteporfin inhibits YAP in 4T1 cells and the corresponding pro-tumorigenic properties of 4T1 TEVs. A) Western blot analysis of 4T1 treated with verteporfin for 24 h. Cell lysates were subjected to western blot analysis by anti-YAP and anti-β-actin antibodies, demonstrating a decrease of YAP protein in a dose-dependent manner. B) Heatmap showing the relative abundance proteins in 4T1 TEVs, specifically those known to induce cell migration and proliferation, directly downstream of YAP/TAZ Hippo signaling pathway. The color code indicates relative abundance, ranging from blue (low abundance) white to red (high abundance). C) Cell proliferation assay of 4T1 cells each treated with different doses of TEVs for 24 h. Proliferation of live cells was measured using a CCK-8 assay, and compared with untreated cells. (n=8 per group) D, E) Transwell matrigel invasion assay. The transwell was coated with matrigel beforehand. 4T1 cells were suspended in media containing 1% FBS and placed in the upper chamber of the transwell, while the lower chamber was loaded with media containing 10% FBS. Cells were treated with TEVs in the upper chamber, migration was observed after 24 h. (D) Representative microscopic images of 4T1 cells that have migrated through the transwell (Crystal violet staining. Magnification, X10). (E) Number of migrated 4T1 cells were counted using ImageJ software.

One-way ANOVA followed by Tukey's posthoc test was calculated using GraphPad PRISM (***p<0.001, ****p<0.0001). Data are presented as the mean±SD.

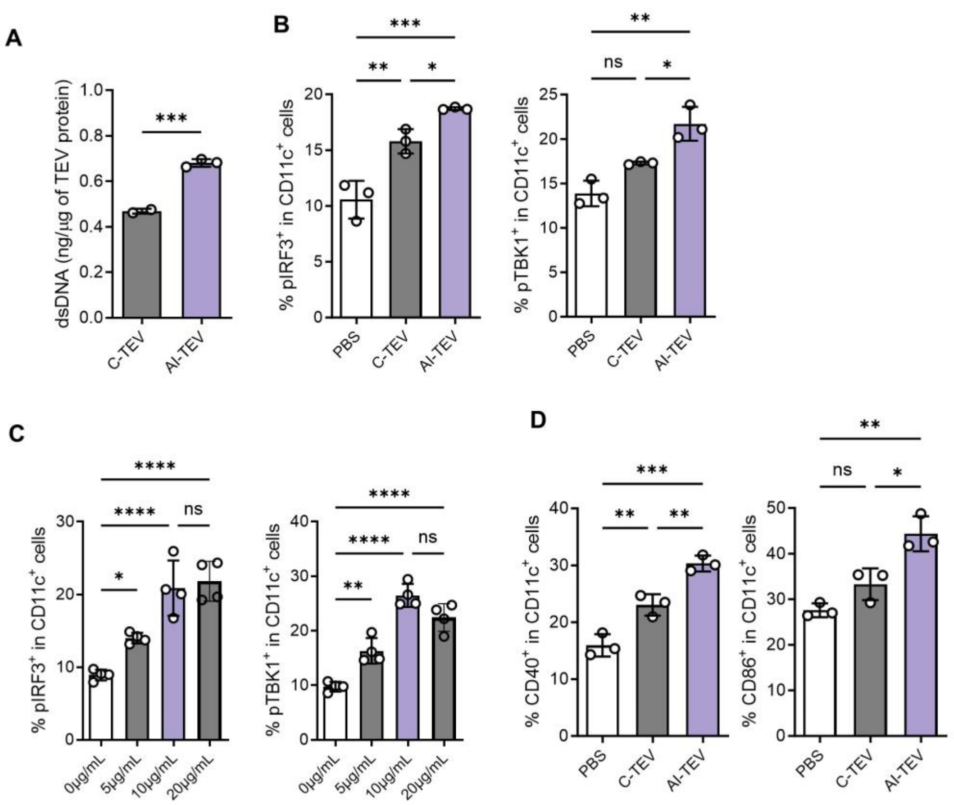


**Figure S4.** TEVs have heightened ability to prime DCs. A) Quantification of dsDNA in MOC2 TEVs using QuantiFluor dsDNA system. B) Flow cytometric analysis of pIRF3 and pTBK1 expression in CD11c$^+$ BMDCs treated with MOC2 TEVs for 48 h. C) Flow cytometric analysis of pIRF3 and pTBK1 expression in CD11c$^+$ BMDCs treated with different doses of E.G7-OVA TEVs for 48 h. D) Flow cytometric analysis of DC maturation markers (CD40, CD86) in CD11c$^+$ BMDCs treated with E.G7-OVA TEVs for 48 h. One-way ANOVA followed by Tukey's posthoc test was calculated using GraphPad PRISM (*p<0.05, **p<0.01, ***p<0.001, ****p<0.0001). Data are presented as the mean±SD.

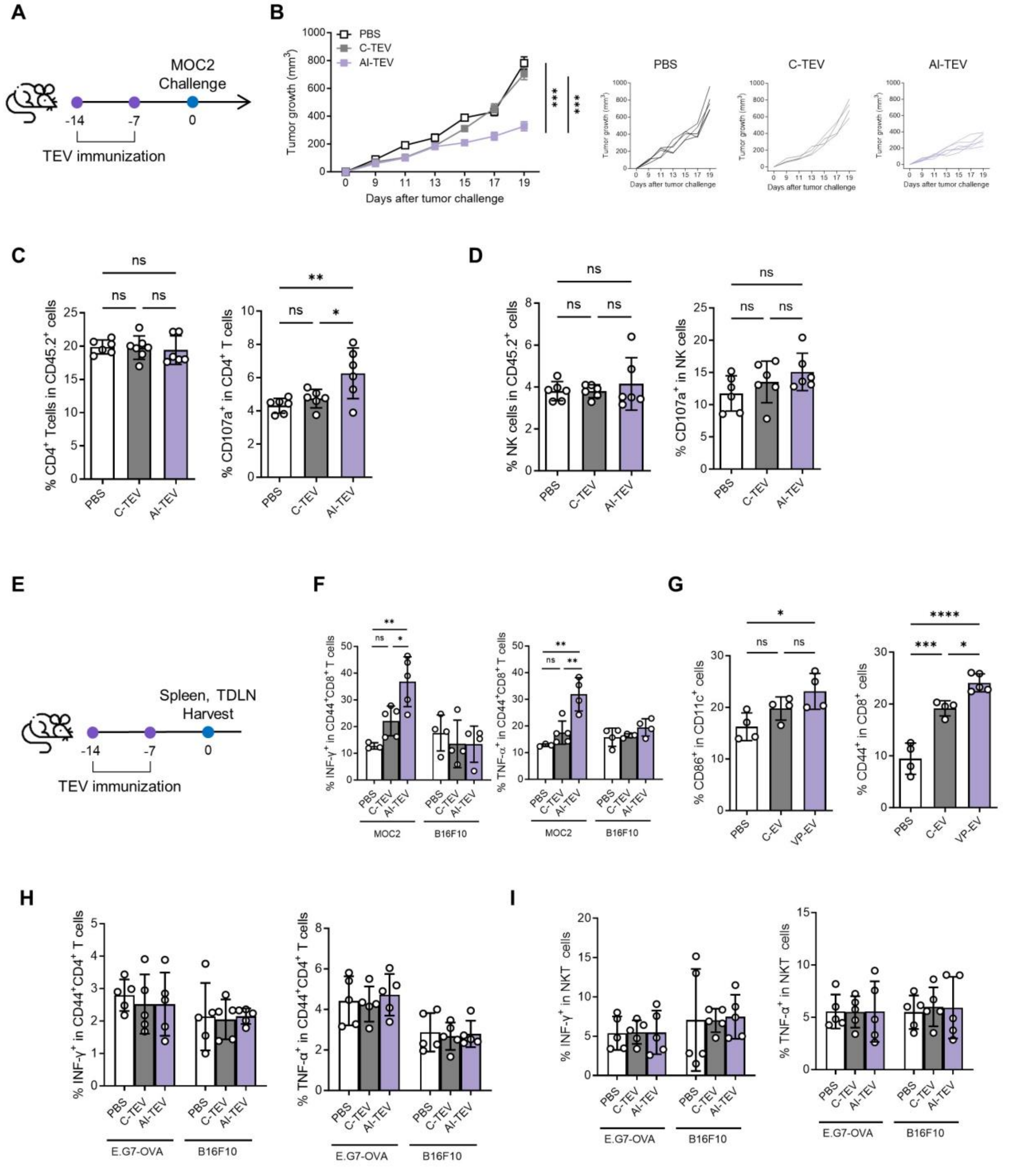


**Figure S5.** AI-TEV induces limited tumor-specific action in CD4$^+$ T cells and NK cells, and is capable of activating tumor-specific immunity against MOC2. A) Prophylactic vaccination schedule of TEVs. B) Average tumor growth curves (n=4-6 per groups) and individual tumor growth curves. C) Portion of CD3$^+$ CD4$^+$ T cells and CD107a$^+$ CD4$^+$ T cells in spleen of vaccinated mice. D) Portion of CD3$^-$ NK1.1$^+$ cells and CD107a$^+$ NK1.1$^+$ cells in spleen of vaccinated mice. E) Scheme of vaccination experiment design for analysis of MOC2-derived TEV-induced immunity. F) Splenocytes were stimulated with UV-irradiated cancer cells for 5 h and analyzed with flow cytometry to identify the percentage of IFN-γ$^+$ and TNF-α$^+$ cells among CD44$^+$CD8$^+$ cells. G) Flow cytometry analyses of the percentage of CD86$^+$ cells among CD11c$^+$ cells and the percentage of CD44$^+$ cells among CD3$^+$CD8$^+$ cells from TDLNs.

H) Splenocytes were stimulated with UV-irradiated cancer cells for 5 h and analyzed with flow cytometry to identify the percentage of IFN-$\gamma^+$ and TNF-$\alpha^+$ cells among $CD44^+$ $CD4^+$ cells. I) Splenocytes were stimulated with UV-irradiated cancer cells for 5 h and analyzed with flow cytometry to identify the percentage of IFN-$\gamma^+$ and TNF-$\alpha^+$ cells among $CD3^+$ $NK1.1^+$ NKT cells.
One-way ANOVA followed by Tukey's posthoc test was calculated using GraphPad PRISM (*p<0.05, **p<0.01, ***p<0.001). Data are presented as the mean±SD.

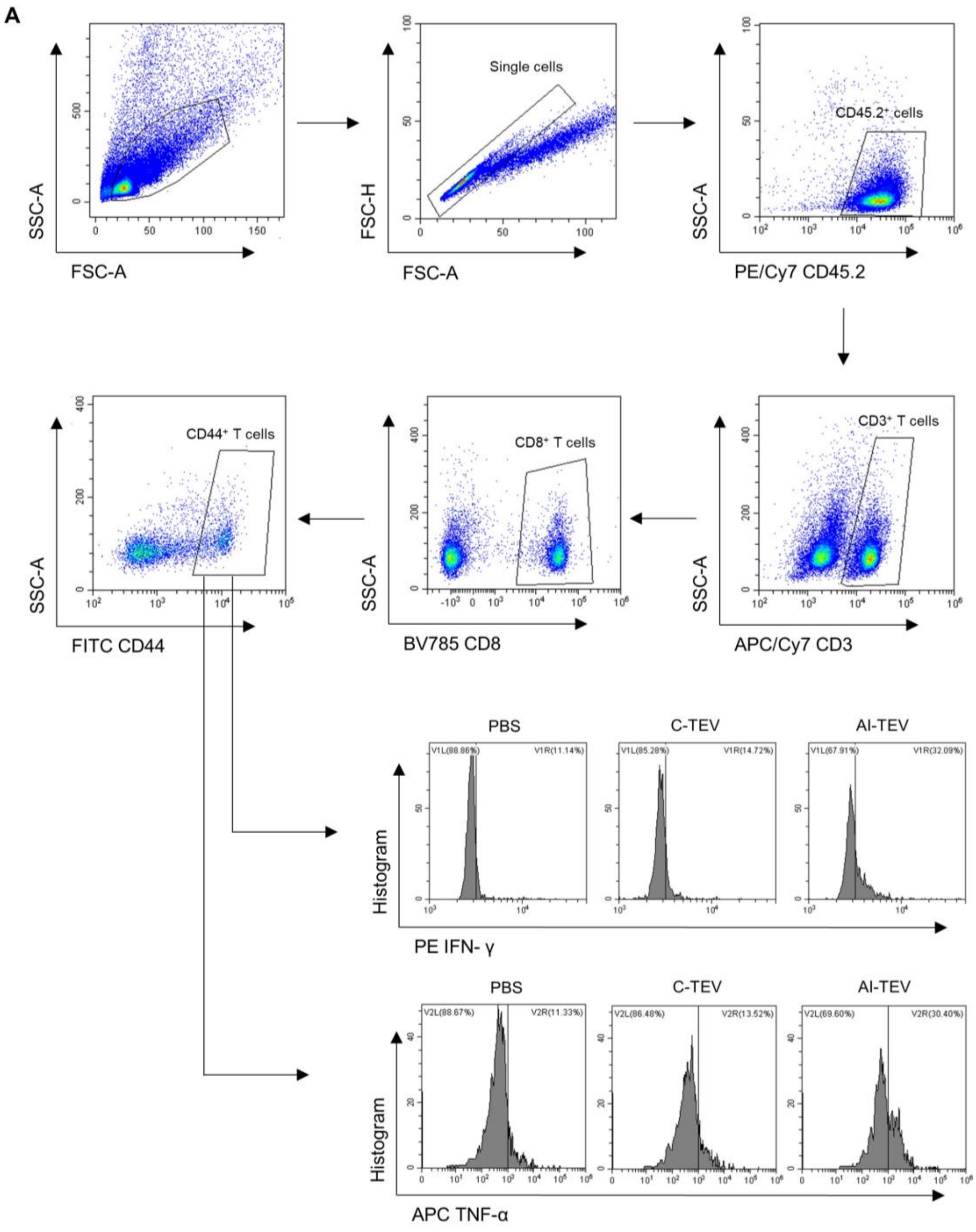


**Figure S6.** Flow cytometry gating strategy for the analysis of tumor cell-pulsed splenocytes. A) Splenocytes were stimulated with irradiated cancer cells, fixed and permeabilized, then analyzed with flow cytometry. Splenocytes were first gated on using FSC-A and SSC-A profiles, doublets were removed using FSC-A and FSC-H. Immune cells were selected by

gating on CD45.2⁺ cells, followed by CD3⁺ T cells, CD8⁺ T cells, and CD44⁺ T cells. Finally, IFN-γ⁺ and TNF-α⁺ cells were identified by gating on CD44⁺ cells.

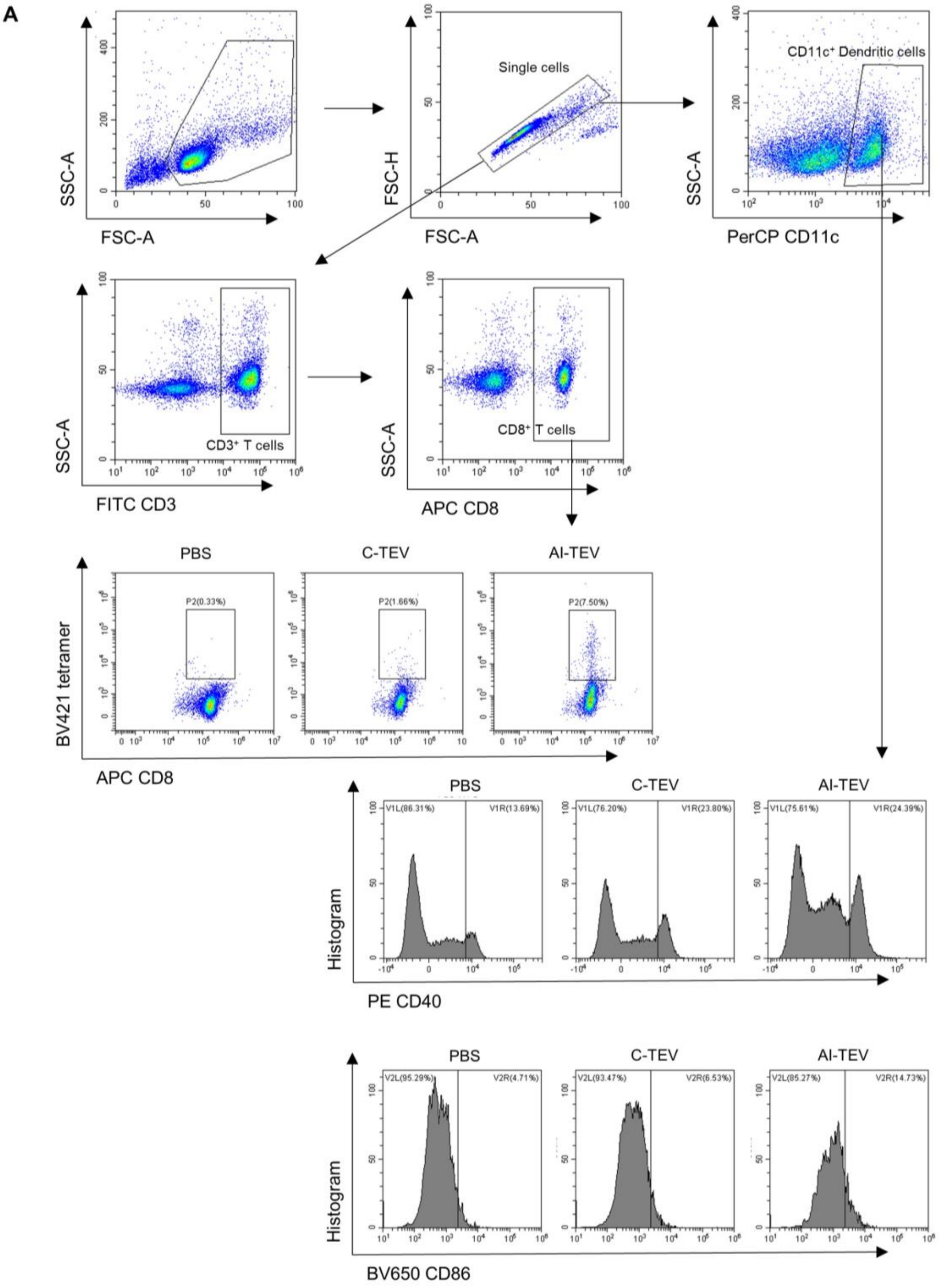


**Figure S7.** Flow cytometry gating strategy for the analysis of TDLNs. A) TDLN cells were gated on using FSC-A and SSC-A profiles, doublets were removed using FSC-A and FSC-H. DC costimulatory molecules CD40 and CD86 were analyzed gated on CD11c⁺ DCs. CD3⁺ T cells were selected, followed by CD8⁺ T cells, among which the percentage of tetramer⁺ CD8⁺ cells were identified.

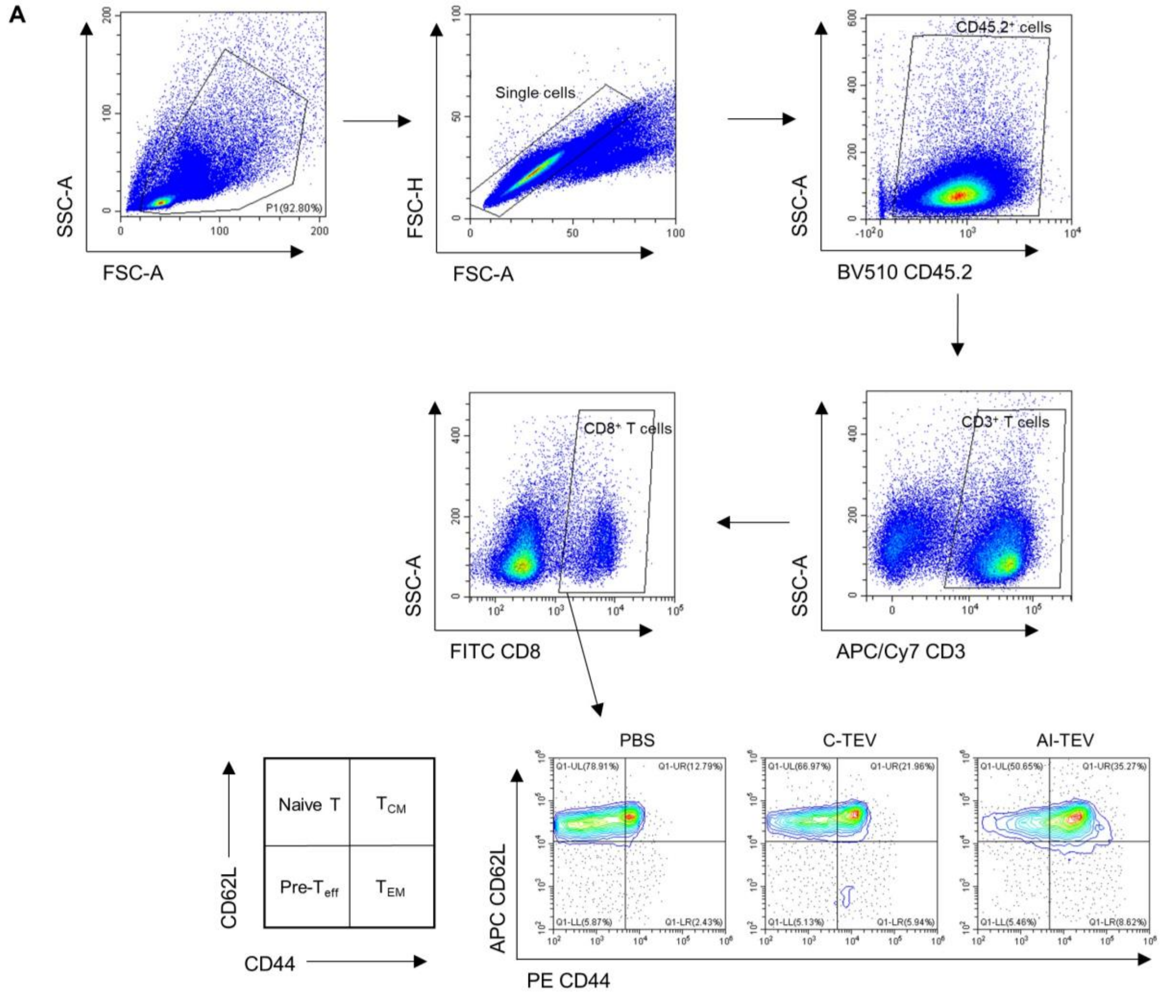


**Figure S8.** Flow cytometry gating strategy for the analysis of memory T cells in spleen. A) Splenocytes were first gated on using FSC-A and SSC-A profiles, doublets were removed using FSC-A and FSC-H. Immune cells were selected by gating on CD45.2$^{+}$ cells, followed by CD3$^{+}$ T cells, CD8$^{+}$ T cells. Memory T cells were identified by the degree of CD44 and CD62L on CD8$^{+}$ T cells.
